# 

**Published:** 1842-07-16

**Authors:** 


### CONTENTS OF No. 29.

Dr. Reynell Coates' Contributions to Surgery, - - - - - - 449
Dr. David H. Tucker's Case of Lumbar Abscess, with Remarks, - - 452
BIBLIOGRAPHICAL NOTICE: Experiments on Kiesteine; with Remarks on its application to the Diagnosis of Pregnancy. An Inaugural Dissertation for the degree of Doctor of Medicine. By Elisha K. Kane, M. D., of Philadelphia, - - - 455
M. W. Wilson's Clinical Reports,
Meningitis, - - - - - - - - - - 459
Apoplexy, - - - - - - - - - - 459
Congestion of the Brain, - - - - - - - 459
Case of Arachnitis, - - - - - - - - - 460
Remarks, - - - - - - - - - - - 461
ANALECTA: Chemical Phenomena of Digestion, - - - - - - 461
Circular Stricture—Enormous Distension of the Bladder, - 462
Complete Obliteration of Urethra—Gangrene of Bladder, - 463
Lateral Operation after Lithotrity in a Child, - - - 463
Foreign body in the Trachea, - - - - - - - 463
Solution of Urinary Calculi, - - - - - - - 463
Cauterisation of the Neck of the Uterus, - - - - 464
Dysmenorrhœa, - - - - - - - - - - 464

